# Genomic Distribution of H3K9me2 and DNA Methylation in a Maize Genome

**DOI:** 10.1371/journal.pone.0105267

**Published:** 2014-08-14

**Authors:** Patrick T. West, Qing Li, Lexiang Ji, Steven R. Eichten, Jawon Song, Matthew W. Vaughn, Robert J. Schmitz, Nathan M. Springer

**Affiliations:** 1 Department of Plant Biology, University of Minnesota, Saint Paul, Minnesota, United States of America; 2 Department of Genetics, University of Georgia, Athens, Georgia, United States of America; 3 Institute of Bioinformatics, University of Georgia, Athens, Georgia, United States of America; 4 Texas Advanced Computing Center, University of Texas-Austin, Austin, Texas, United States of America; Duke University, United States of America

## Abstract

DNA methylation and dimethylation of lysine 9 of histone H3 (H3K9me2) are two chromatin modifications that can be associated with gene expression or recombination rate. The maize genome provides a complex landscape of interspersed genes and transposons. The genome-wide distribution of DNA methylation and H3K9me2 were investigated in seedling tissue for the maize inbred B73 and compared to patterns of these modifications observed in *Arabidopsis thaliana*. Most maize transposons are highly enriched for DNA methylation in CG and CHG contexts and for H3K9me2. In contrast to findings in *Arabidopsis*, maize CHH levels in transposons are generally low but some sub-families of transposons are enriched for CHH methylation and these families exhibit low levels of H3K9me2. The profile of modifications over genes reveals that DNA methylation and H3K9me2 is quite low near the beginning and end of genes. Although elevated CG and CHG methylation are found within gene bodies, CHH and H3K9me2 remain low. Maize has much higher levels of CHG methylation within gene bodies than observed in *Arabidopsis* and this is partially attributable to the presence of transposons within introns for some maize genes. These transposons are associated with high levels of CHG methylation and H3K9me2 but do not appear to prevent transcriptional elongation. Although the general trend is for a strong depletion of H3K9me2 and CHG near the transcription start site there are some putative genes that have high levels of these chromatin modifications. This study provides a clear view of the relationship between DNA methylation and H3K9me2 in the maize genome and how the distribution of these modifications is shaped by the interplay of genes and transposons.

## Introduction

Cytosine DNA methylation is a chromatin modification involved in many cellular processes including regulating gene expression and silencing repeat sequences and transposons. In plants, DNA methylation occurs in both symmetrical (CG and CHG, where H is A, T, or C) and asymmetrical (CHH) sequence contexts, where DNA methylation in each of the three contexts is thought to be regulated separately [1]. The symmetrical methylation found in CG or CHG contexts can be maintained via methylation of the hemi-methylated molecule generated by DNA replication. In contract, CHH methylation requires continual targeting of *de novo* methyltransferases that do not require hemi-methylation of the target.

Much of our understanding about DNA methylation machinery and mechanisms in plants is based on research in *Arabidopsis thaliana*. In *Arabidopsis*, CG, CHG, and CHH methylation are highly enriched within transposable elements, repeat sequence, and the pericentromeric region [2]–[3]. CG methylation is largely maintained by the MET1 enzyme [1]. CHG methylation is largely attributed to the CMT3 chromomethylase gene [1]. The proper maintenance of CG and CHG methylation, particularly in heterochromatic regions, requires the chromatin remodeler DDM1 [4]–[5]. The targeting of chromomethylases involves binding to nucleosomes marked by histone 3 lysine 9 dimethylation (H3K9me2) [6]. There is evidence for interdependence between CHG methylation and H3K9me2 such that if either modification is lost, both show genome wide depletion [7]–[9]. There are two different pathways for CHH methylation in *Arabidopsis* [1], [5], [9]–[10]. The RNA-directed DNA methylation (RdDM) pathway requires the DRM methyltransferases and involves PolIV, PolV and other components [1], [9]. The other pathway utilizes the CMT2 methyltransferase and likely requires DDM1 and H3K9me2 methylation [5], [10].

Although the genetic and genomic resources available for *Arabidopsis* have provided substantial opportunities to understand DNA and histone methylation in plants, the *Arabidopsis* genome may not provide a good model for many of the crop genomes. *Arabidopsis* have very few transposons that are mostly clustered in pericentromeric regions or other heterochromatic knobs [11]. The *Zea mays* genome is ∼20 fold larger than the *Arabidopsis* genome and exhibits a complex arrangement of transposons and genes that is observed in many plant species. The majority of maize genes are flanked by transposons [12]–[14] and the chromatin landscape of maize is much more diverse than that of the segregated *Arabidopsis* genome [15]–[16]. Profiling of DNA methylation by methyl-filtration sequencing, restriction analyses or methylated DNA immunoprecipitation (meDIP) has revealed that DNA methylation is enriched over transposons and generally lower over genes [14], [16]–[21]. Several studies have reported whole-genome bisulfite sequencing (WGBS) for maize [22]–[24]. CG and CHG methylation are highly enriched over transposons and repeat sequences and depleted near genic space [22]–[23]. CHH methylation on the other hand does not correlate with CG or CHG methylation, is depleted over repeat sequences, and is enriched near the start and end of genes [22]. Cytological analysis of histone modifications in maize revealed that H3K9me2 was localized throughout the chromosome for pachytene chromosomes and was not particularly enriched at pericentromeric or knob heterochromatin [25]–[26]. However, there is evidence for enrichment of H3K9me2 at transposon sequences in maize that are generally considered to be heterochromatic [27]–[28].

The relationship between DNA methylation and other chromatin modifications has not been looked at in great detail in maize. Here we combine WGBS with H3K9me2 ChIP-seq to assess the relationship between DNA methylation and H3K9me2 throughout the maize genome. In comparison to *Arabidopsis* we find lower levels of CHH methylation and a distinct relationship of H3K9me2 with CHH methylation patterns. The analysis of different sub-families of transposable elements reveals distinct patterns of CHH and H3K9me2 enrichment for different families. Maize genes contain CHG methylation within the gene body and this is partially attributable to the presence of transposons in introns of >10% of maize genes. The presence of heterochromatin transposons within genes does not appear to restrict expression of these genes. We also identified a subset of maize genes with high levels of CHG methylation or H3K9me2 over the transcription start site (TSS) and find that the majority of these genes are not expressed throughout maize development.

## Methods

### Bisulfite Sequencing

Genomic DNA was isolated from the third leaf of 14-day after planting seedling from the B73 inbred line. Samples were fragmented and ligated with TruSeq-methylated adapters. Bisulfite conversion was performed on five hundred nanograms of adaptor-ligated DNA using the MethylCode bisulfite conversion kit (Life Technologies) according to manufacturer’s guidelines. Converted DNA was split into four reactions and amplified using Pfu Turbo Cx DNA polymerase (Agilent) for four cycles and subsequently pooled. Libraries were sequenced on the HiSeq 2000 (Illumina) for 100 cycles, paired end. Sequencing reads (SRA accession SRP022569) were processed to identify and filter poor 3′ quality and incomplete conversion. Sequences were aligned to the B73 reference genome (AGPv2) using the Bismark aligner (v0.7.2; [29]) under the parameters (-n 2, -l 50). Methylated cytosines were extracted from aligned reads using the Bismark methylation extractor under standard parameters. The proportion of CG, CG, and CHH methylation was determined as weighted methylation levels [30] in 100bp non-overlapping windows across the genome.

### H3K9me2 ChIP-seq

H3K9me2 profiling was performed on three replicates of B73 seedling using antibodies specific for H3K9me2 (#07-441) purchased from Millipore (Billerica, USA) according to manufacturer’s recommendations as described in Eichten et al. [27]. For ChIP-seq, adapters were ligated to replicates using one of two protocols. In the first, TruSeq-methylated adapters were ligated to the B73 DNA fragments according to the NEBNext DNA Library Prep protocol. In the second, adapters were ligated using the Nextera DNA sample Preparation Kit. Both samples were sequenced to greater than 100 million reads on the HiSeq 2000 (Illumina), single end. Sequencing reads (SRA accession SRP043372) were analyzed using FastQC (http://www.bioinformatics.babraham.ac.uk/projects/fastqc/) to identify and filter poor 3′ quality reads. Sequenced reads were aligned to the B73 reference genome (AGPv2) using Bowtie under standard parameters. Duplicate reads from both the NEBNext and Nextera libraries were removed using SAMtools [31] and the samples were merged into one library. The level of H3K9me2 was described as the sum of the intersecting H3K9me2 ChIP-seq reads over 100bp windows across the genome. Intersecting 100 bp windows and H3K9me2 reads were determined using BEDtools [32]. 100 bp windows with significant H3K9me2 were defined as having a sum of reads greater than one standard deviation above the average sum.

### RNA-seq and Expression Analysis

RNA isolated from 14-day after planting leaf tissue of the B73 inbred line was prepared for sequencing at the University of Minnesota Genomics Center using the TruSeq library preparation protocol (Illumina). Three independent replicates were included. Libraries were sequenced on the HiSeq 2000. Over 10 million 50bp read pairs were generated for each library. Raw reads (SRA accession SRP018088) were filtered to eliminate poor-quality reads using CASAVA 1.8 (Illumina). High-quality reads were then passed to Trim_glore (http://www.bioinformatics.babraham.ac.uk/projects/trim_galore/) to trim poor bases from 3′ end of the sequences, to remove adapters and to filter very short reads resulted from base and adapter trimming. This was run under the pair-end reads mode using standard parameters. Reads that passed quality control were first mapped to the Filtered Gene Set (ZmB73_5a, FGS), and unmapped reads were realigned to the maize reference genome (AGPv2) using TopHat [33] under standard parameters. Only reads that are mapped uniquely to the genome were kept and used to calculate transcript abundance. The number of read pairs that are mapped to each gene were developed using “BAM to Counts” within the iPlant Discovery Environment (www.iplantcollaborative.org). The ‘Reads count per kilobase per million mapped’ (RPKM) value was calculated and averaged over the three biological replicates to represent the expression level of each filtered gene. Those expression levels were used to group the genes into five categories: not expressed, and four categories with equal number of expressed genes in B73 seedling tissues. The proportion in each of the five categories were determined for genes that were identified to have specific features, e.g., with >1000 bp TE, having high H3K9me2 or CHG in promoters.

### Analyzing H3K9me2 and DNA methylation

To analyze the correlation between non-CG methylation (CHG and CHH) and H3K9me2, all the 100 bp windows that have data on all three marks were grouped based on the levels of either CHG or CHH. CHG levels were equally split into 10 groups from 0% to 100%. CHH levels were split into 9 groups, 5 groups from 0 to 5% by an increase of 1%, 3 groups from 5% to 20% by an increase of 5%, and a group of >20%. The CHG and CHH groups were cross-tabulated to give a total of 90 combinations (10 CHG groups * 9 CHH groups), and the average H3K9me2 levels were calculated for each combination and were shown as a heatmap.

The average DNA methylation and H3K9me2 levels for each transposon sub-family was calculated. The classification of transposon sub-families were based on Maize TE Consortium [34] and the study of Eichten et al. [27]. The 100 bp windows that overlap or fall within each transposon sub-family were identified using intersectBed from the BEDtools package. Those windows were used to calculate the average values for both DNA methylation and H3K9me2 using R. We also calculated the mean level of DNA methylation and H3K9me2 in the flanking regions of each transposon sub-family. Briefly, 100 bp windows that overlap with the regions that are 900–1000 bp away from a transposon were identified, and used to get the mean DNA methylation and H3K9me2 for both the upstream and downstream flanking regions.

### Analysis of genes with TEs and genes with high CHG/H3K9me2 in promoters

Genes with high CHG or H3K9me2 in their promoter region were identified by assessing the levels of CHG or H3K9me2 in the 100 bp window that overlaps the transcription start site. Genes with greater than 88.5% CHG methylation (top 10% of all CHG values) in the promoter region were defined as having high CHG over the promoter region and genes with greater than 2 standard deviations of H3K9me2 reads above the genome wide average in the promoter region were defined as having high H3K9me2 over the promoter region.

### Relative distance line plots

To plot DNA methylation or H3K9me2 levels over transposons and their flanking regions, we first determined the distance between the 100 bp windows and transposons from the Maize TE Consortium (ZmB73_5b). Windows upstream of the transposable elements were given a negative distance value and windows downstream a positive distance value. We then identified the closest transposon to each 100 bp window, and kept those windows that are located within the transposons or the 1000 bp flanking regions on either side. For windows overlapping or within transposons, the normalized distance across the element on a scale of 1 to 1000 was determined. The scaled 1000 bp element, together with 2000 bp flanking regions, were then divided into 60 equal bins, 20 bins each for the 1000 bp upstream region, the scaled 1000 bp element, and the 1000 bp downstream region. The average methylation levels of the bins were then determined and plotted on a line graph in R.

### Absolute distance line plots

The absolute distance line plots consist of two parts, the 5′ plot and the 3′ plot, each of which contains 5 kb genomic segments. The 5′ plot contains 2 kb upstream regions of the transcriptional start site (TSS) and 3 kb genic sequences from TSS. The 3′ plot contains 2 kb downstream sequences from the transcriptional termination site (TTS) and 3 kb genic sequences from the TTS. For genes that are less than 3 kb, the actual gene size were used, which means less than 5 kb regions will be used. In other words, the further into a gene, the less number of genes will be included in those plots. To make these plots, the physical distance between genes and nearby 100 bp windows was determined. For the 5′ plot, this distance was determined to be the physical distance between the mid-point of the 100 bp window and the TSS. While for the 3′ plot, it was calculated as the physical distance between the mid-point of each window and the TTS. Windows that are falling within the respective 5 kb genomic regions of a gene were kept for downstream analysis. For each plot, the 5 kb regions were then divided into 100 equal bins, and the average methylation level for each bin across all genes were calculated using R. Finally, the averaged methylation level was plotted against the center of each bin using R.

## Results

**Figure 1:**
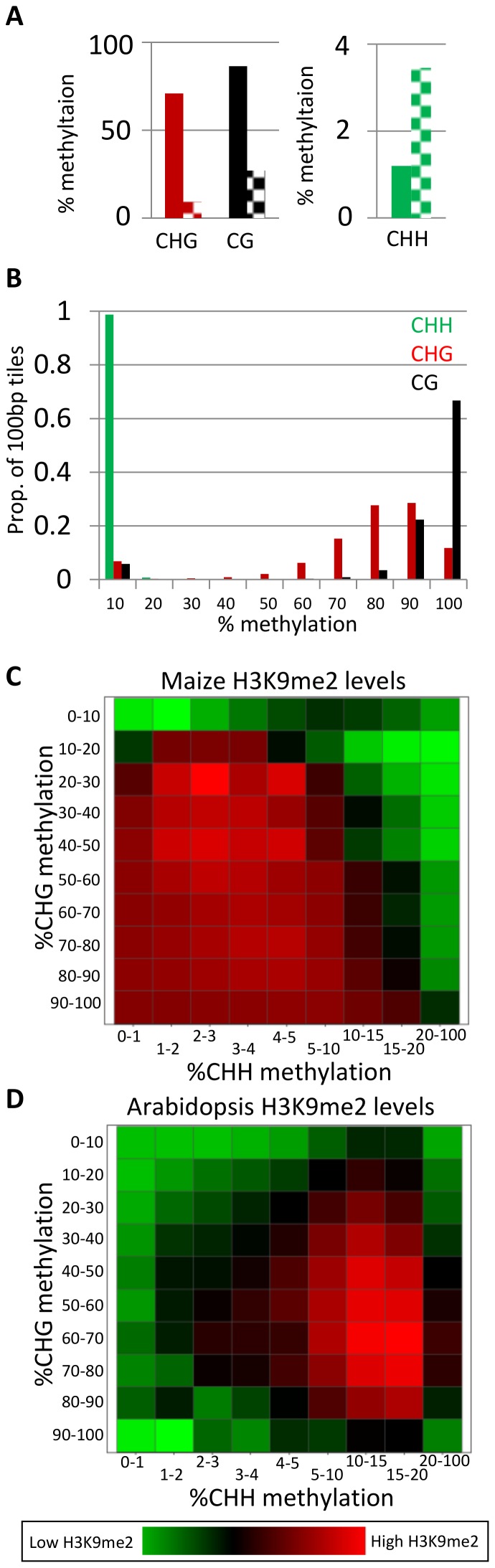
Genome-wide levels of DNA methylation and H3K9me2. (A) Average genome-wide DNA methylation levels in CG, CHG, and CHH sequence contexts. These numbers refer to the average DNA methylation level for all cytosines within this sequence context. Solid bars are maize DNA methylation and dashed bars are *Arabidopsis* DNA methylation (data from [34]). (B) The proportion of 100 bp tiles across the maize genome containing different levels of DNA methylation is shown. (C–D) The average read number for H3K9me2 ChIP-seq was determined for 100 bp tiles in maize (C) or *Arabidopsis* (D) having varying levels of CHG and CHH methylation and is shown using a heat map to illustrate relative enrichment.

To investigate the distribution of DNA methylation and H3K9me2 in the maize genome we performed WGBS and H3K9me2 ChIP-seq on leaf tissue of B73 maize seedlings (Figure S1A). The same tissue was also used to perform RNA-seq in order to compare the distribution of these chromatin modifications relative to gene expression (Figure S1A). On a genome-wide scale, the level of CHH methylation is very low with 1.2% of total CHHs methylated whereas CHG and CG methylation are relatively high with 70.9% and 86.4% methylation respectively (Figure 1A). Similar to maize, the *Arabidopsis* genome (SRA accession SRA035939) also contains more CG and CHG methylation than CHH methylation (Figure 1A). The comparison of maize and *Arabidopsis* reveals ∼2-fold higher levels of CG and CHG in the maize genome and ∼2-fold lower levels of CHH methylation in maize (Figure 1A). The observation of higher genome-wide CG and CHG methylation in maize may simply reflect the higher transposon content of the maize genome.

The CG, CHG and CHH methylation levels for 100 bp tiles of the maize genome were assessed. The vast majority of regions show less than 10% CHH methylation with only 1.3% of regions exhibiting >10% CHH methylation (Figure 1B). In contrast, the majority of tiles exhibit high levels of CG or CHG methylation, similar to analyses of other maize tissues [22], [23]. However, a small portion (5–10%) of the maize genome exhibits less than 10% CG or CHG methylation. The analysis of 100 bp tiles located in genes or transposons reveals that the majority of 100 bp tiles with low (<10%) CG (72%) or CHG (74%) methylation are located within genes (Figure S1B–C). This supports the utility of the methyl-filtration sequencing that provided targeted sequencing for these unmethylated regions [35]. The levels of CHH methylation are quite low both in genes and within transposons (Figure S1D). The distribution of sequencing depth for 100 bp tiles reveals that genic sequences tend to have much lower levels of H3K9me2 than TEs (Figure S1E).

There is growing evidence that histone modifications, in particular H3K9me2, can play a role in targeting DNA methylation in *Arabidopsis*, especially in the CHG and CHH contexts [6], [10], [36]. We assessed how H3K9me2 levels were associated with CHG and CHH methylation throughout the maize and *Arabidopsis* (SRA accession GSM124393) genomes (Figure 1C–D). Maize exhibits high levels of H3K9me2 whenever there is >20% CHG methylation and less than 10% CHH methylation (Figure 1C). In contrast, H3K9me2 is associated with higher levels of CHH methylation in *Arabidopsis* (Figure 1D).

### DNA methylation and H3K9me2 profiles over maize transposons

**Figure 2:**
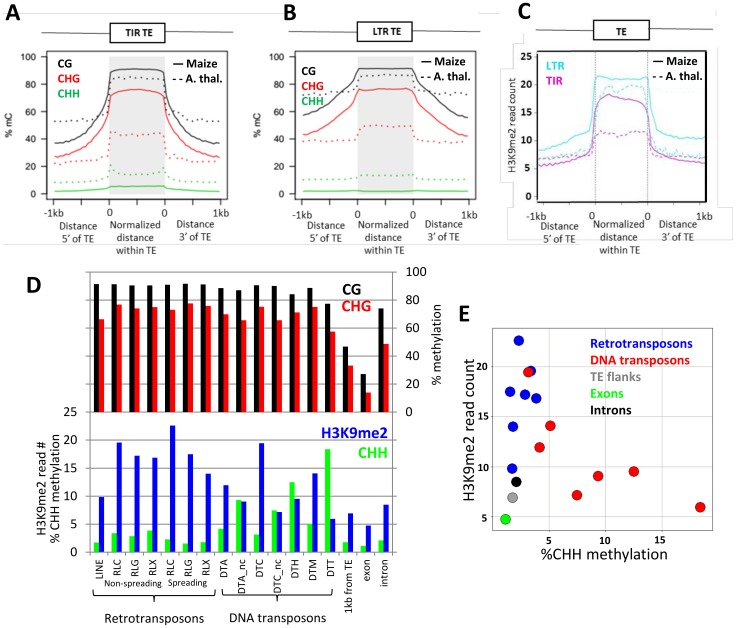
Enrichment of DNA methylation and H3K9me2 over transposable elements. (A–B) Relative distance line plots of DNA methylation over transposable elements in maize and *Arabidopsis*. The plot in A shows the average enrichment for each type of DNA methylation of DNA transposons containing terminal inverted repeats (TIRs). The colors of the lines indicate the context of DNA methylation (black-CG; red-CHG; green-CHH) and type of line indicates the species (solid = maize; dashed = *Arabidopsis*). In (B) similar plots are shown for long terminal repeat (LTR) retrotransposons. (C) Plots of H3K9me2 abundance are shown for DNA transposons (purple) and retrotransposons (blue) in both maize (solid lines) and *Arabidopsis* (dashed lines). (D) The average level of DNA methylation or H3K9me2 is plotted for different sub-classes of transposable elements. The retrotransposons are divided into LINEs, RLG (gypsy-like), RLC (copia-like) and RLX (LTR elements of unknown class). The RLG, RLC and RLX elements are all split according to whether they exhibit evidence for spreading of H3K9me2 or DNA methylation in flanking regions as defined in Eichten et al [27]. The DNA transposons are divided into five major families (DTA (*hAT*), DTC (*CACTA*), DTH (*PIF/Harbinger*), DTM (*Mutator*) and DTT (*Tc1/Mariner*)) and for two of these there are large families of “non-coding” elements that are indicated as “-nc”. The last three bars indicate the average levels for each modification 1 kb away from TEs, within exons or within introns. (E) The relative levels of H3K9me2 and CHH methylation are shown for each sub-family of transposons. The levels for TE flanking regions, exons and introns are also shown.

DNA methylation and H3K9me2 are frequently enriched over transposable element sequences. The profile of DNA methylation and H3K9me2 over maize and *Arabidopsis* DNA transposons or retrotransposons was compared (Figure 2A–C). The maize data are from the current study whereas the *Arabidopsis* DNA methylation data is obtained from Schmitz et al. [37] and the *Arabidopsis* H3K9me2 data were obtained from Stroud et al., [10]. In both maize and *Arabidopsis*, the level of CG and CHG methylation is markedly higher in TEs compared to flanking regions. However, there is more evidence for spreading of this DNA methylation to the flanking regions in maize than in *Arabidopsis* as evidenced by the slope of the profile in maize rather than the sharp drop seen in *Arabidopsis*. In addition, the abundance of CHG methylation is substantially higher for maize than for *Arabidopsis* at both class I (LTR elements - RNA intermediate) and class II (TIR elements - DNA intermediate) transposons. While *Arabidopsis* class II transposons exhibit enrichment for CHH methylation near the TIRs (the bumps in the CHH profile at the beginning and end of TIR elements) this enrichment is not noted in maize. H3K9me2 is enriched over both TIR DNA transposons and LTR retrotransposons in both maize and *Arabidopsis* (Figure 2C). The enrichment of H3K9me2 is more pronounced over LTR elements as compared to TIR elements (Figure 2C).

The profiles of DNA methylation and H3K9me2 were examined for a number of sub-families of maize transposons using the classifications from the maize genome annotation [14], [34]. These include LINE elements, nine sub-types of LTR elements and seven sub-types of TIR elements (full profiles for each class are available in Figure S2). The level of DNA methylation or H3K9me2 was determined within each family and is compared to the average levels observed in flanking regions (1 kb from the elements) or exons (Figure 2D). The levels of CG and CHG methylation are uniformly high for all sub-types of transposons (Figure 2D). The levels of CHH methylation show some unusual trends. The LTR families tend to have quite low levels of CHH methylation but the families that exhibit spreading of heterochromatin to flanking sequences [27] have lower levels of CHH than families that do not exhibit spreading. Some of the TIR families are marked by quite high levels of CHH methylation (Figure 2D) and these same families exhibit elevated CHH in other maize tissues as well [22]. There are also significant differences in the relative levels of H3K9me2 over different families. In general, the LTR families all have quite high levels of H3K9me2 while some of the TIR families have quite low levels of H3K9me2 (Figure 2E). The families with the highest levels of CHH methylation tend to have the least enrichment for H3K9me2.

### DNA methylation and H3K9me2 profiles over maize genes

**Figure 3:**
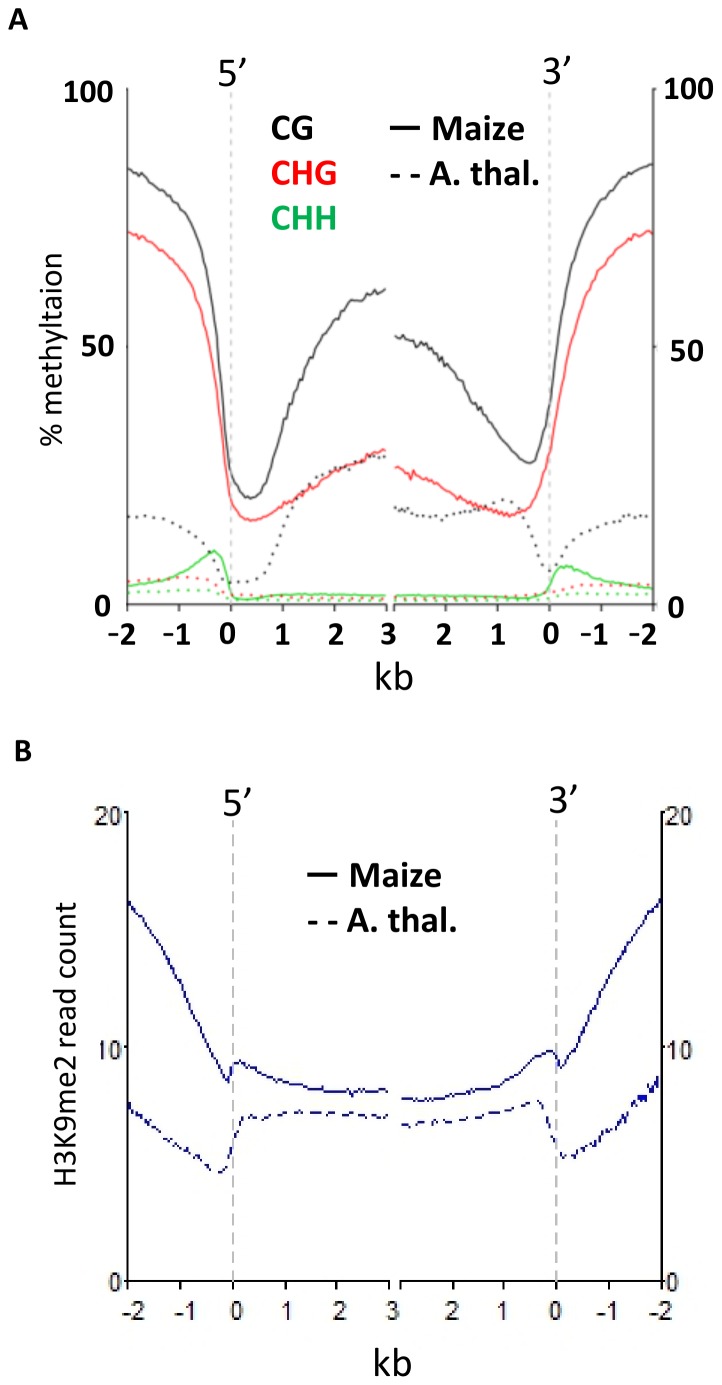
Absolute distance line plots of DNA methylation and H3K9me2 over genic space in maize and *Arabidopsis*. (A) Maize and *Arabidopsis* genes were aligned at the 5′ and 3′ ends and average DNA methylation are plotted for the regions beginning 2 kb from the gene to the regions within the gene. The vertical dashed lines represent the 5′ transcriptional start site (TSS) and 3′ transcriptional termination site (TTS). (B) A similar plot is used to show average levels of H3K9me2 for maize and *Arabidopsis* genes.

The profile for each type of DNA methylation and H3K9me2 over genes was compared in maize and *Arabidopsis* (Figure 3A–B). The patterns observed in maize and *Arabidopsis* are somewhat similar but there are a number of differences. The level of CG and CHG DNA methylation in the 2 kb upstream of the transcription start site (TSS) or 2 kb downstream of the transcription termination site (TTS) is much lower in *Arabidopsis* than in maize. This is likely a result of many maize genes being flanked by transposons sequences and exemplifies the different chromatin environment surrounding maize genes compared to *Arabidopsis* genes. Maize genes also tend to be closely flanked by regions of elevated CHH methylation, termed CHH islands by Gent et al. [22] and also noted by Regulski et al. [23]. Both *Arabidopsis* and maize genes exhibit increased levels of CG methylation in the middle of the transcribed regions relative to the regions near the TSS and TTS. Maize gene bodies also contain noticeable CHG methylation whereas this chromatin modification is not observed within *Arabidopsis* gene bodies. The H3K9me2 profiles reveal depletion in the regions immediately preceding or following the TSS and TTS in both maize and *Arabidopsis* (Figure 3B) that could reflect nucleosome free regions.

The profile of DNA methylation over maize genes is influenced by several comparative genomic attributes. The full set of potential annotated maize genes are classified as a working gene set (WGS; n = 110,028) and a subset (n = 39,656) are classified as the filtered gene set (FGS). The FGS genes are a subset of putative genes with more evidence for functionality (full-length cDNA, homology to coding sequence in other species) whereas the WGS genes may include pseudo-genes, misannotated transposable elements, or gene fragments. The CG and CHG DNA methylation profile for the FGS genes shows much greater reductions in DNA methylation levels at the TSS and TTS (Figure S3A). In contrast, the FGS genes are marked by higher levels of CHH methylation in the regions immediately preceding or following the transcribed region. The H3K9me2 levels are more strongly reduced for FGS genes than WGS genes. The FGS genes can be split into a group with retained syntenic positions relative to sorghum and rice and genes that have inserted into new genomic positions. The inserted genes have much higher levels of CHG and CG methylation both within introns and exon (Figure S3B). The ancient tetraploid nature of the maize genome resulted in many examples of retained paralogs that have been assigned to two subgenomes based on preferential fractionation and expression [38]. However, we did not find evidence for differences in DNA methylation profiles for retained duplicates that are present within both subgenomes (Figure S3C). This suggests that DNA methylation does not play a critical role in distinguishing the sub-genomes but there can be differences in DNA methylation levels at specific pairs of retained duplicates. Similar findings have been reported in maize [21], soybean [39] and brassica [40].

### CHG methylation and H3K9me2 within maize gene bodies is due to presence of transposons in maize introns

**Figure 4:**
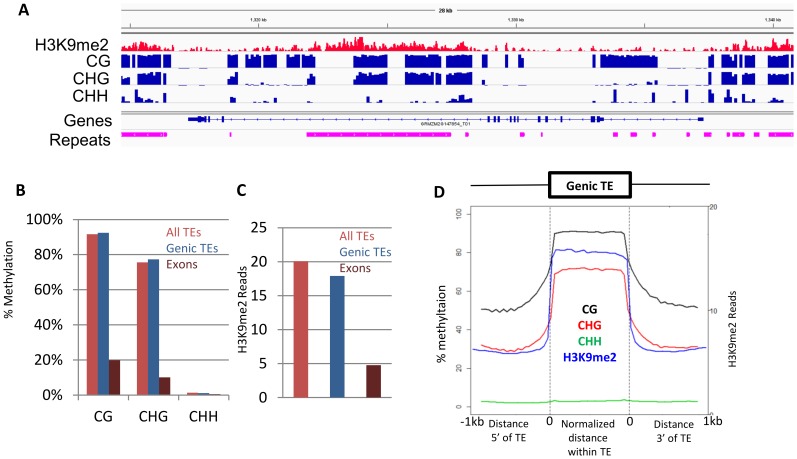
DNA methylation and H3K9me2 levels of genic transposons. (A) An example of a transposable element insertion located within an intron of a maize gene is shown using Integrated Genome Viewer [48]. The H3K9me2 ChIP-seq read count is shown in red. The levels of CG, CHG and CHH methylation (per 100 bp tile) are shown in blue. (B) The average CG, CHG and CHH DNA methylation levels are shown for all TEs (orange), genic TEs (blue) and exons (maroon). (C) The average H3K9me2 read counts are shown for the same regions. (D) The profile of DNA methylation and H3K9me2 over genic TEs is shown.

The presence of CHG methylation within maize gene bodies was unexpected as this chromatin modification is not often found in *Arabidopsis* genes. Separately plotting the levels of CHG for intron and exon regions reveals that much of this gene-body CHG methylation is derived from introns rather than exons (Figure S3). A recent study noted that a small number of *Arabidopsis* genes contained introns with elevated levels of CHG methylation, often due to the presence of transposons within these introns [41]. We found that 4156 of the 39656 FGS maize genes contain transposons >1,000 bp inserted within introns (examples in Figure 4A and Figure S4). If these transposons within maize genes are masked then we find that the level of CHG and H3K9me2 methylation within maize genes drops substantially (Figure S5A–D).

**Figure 5:**
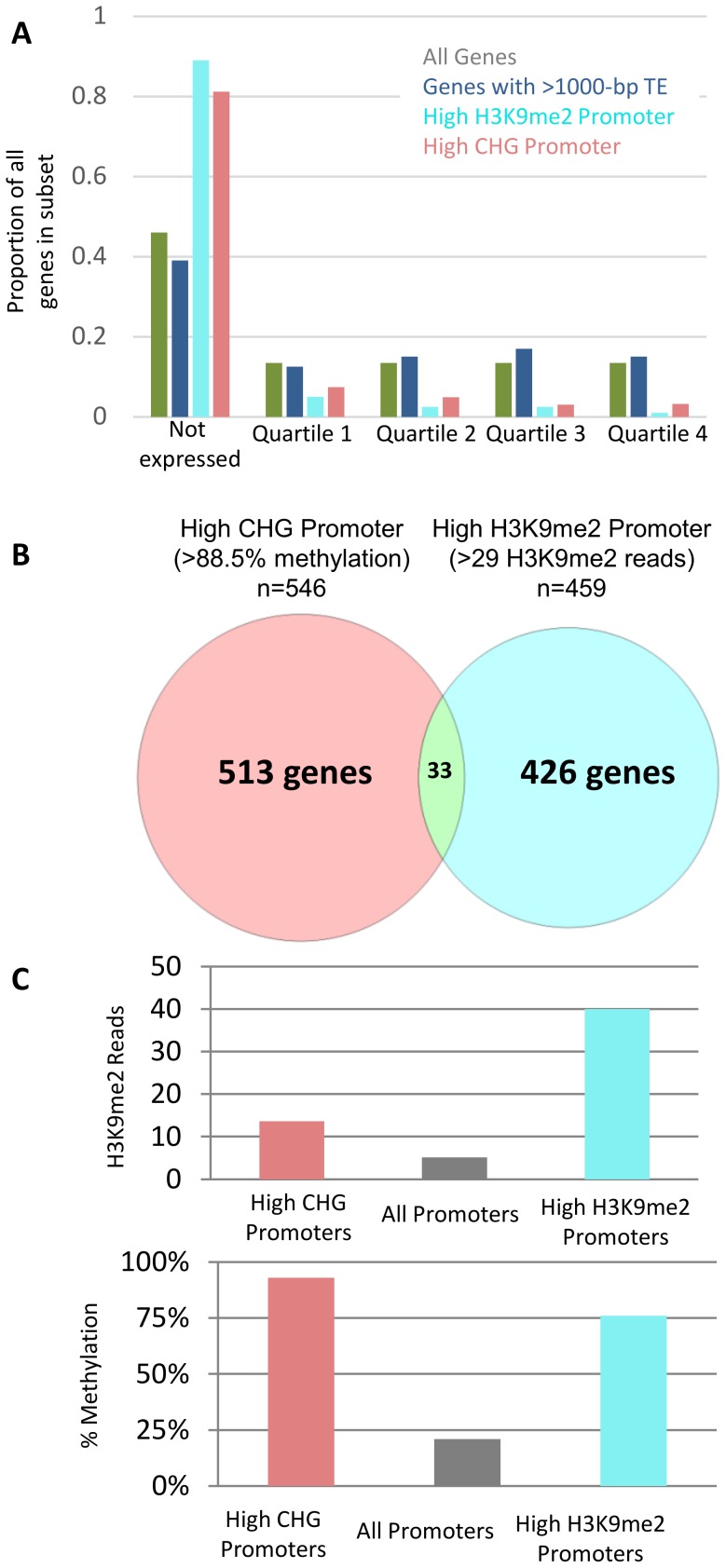
Effect of high CHG methylation or H3K9me2 over promoter on expression. (A) All maize genes were grouped into 5 groups: a subset of genes not expressed and then of the expressed genes, four quartiles of equal size increasing in expression level from 1 to 4 (gray). The relative proportion of genes containing TE insertions (dark blue), genes with high H3K9me2 (light blue) or high CHG in promoters (pink) in these five groups is shown. (B) Genes containing high levels of CHG or H3K9me2 over the transcription start site were identified and the overlap is shown (full list of genes in each category is available in Table S1). (C) Relative level of CHG methylation or H3K9me2 in each subset of promoter regions.

The transposons insertions within genes were further characterized to understand whether the chromatin of these transposons differed from the chromatin at non-genic insertion (Figure 4B–C). The level of DNA methylation or H3K9me2 for these transposons inserted within genes is similar to transposons located outside of genes and is much higher than the levels observed in exons (Figure 4B–C). The profile of DNA methylation and H3K9me2 for these transposons inserted within genes reveals very stark boundaries between the transposon and the flanking sequence (Figure 4D) providing evidence for precise targeting of these modifications and lack of spreading for the chromatin modifications to flanking exon or intron sequences. The presence of large transposons that are marked by CHG and H3K9me2 within maize FGS genes may pose a problem for gene expression. The relative expression level of each gene was assessed and all expressed genes were assigned to quartiles. The presence of transposons within genes did not result in more examples of genes without expression and was not associated higher or lower expressed genes (Figure 5A). The majority of long TE insertions within genes are class I LTR elements (Figure S5E). A comparison of the frequency for each class of element in the whole genome to the frequency of each class within genes reveals enrichment for LINE elements within genes (Figure S5E).

### Genes with elevated CHG or H3K9me2 near TSS

The average levels of CHG and H3K9me2 are quite low near the transcription start site of maize genes (Figure 3A–B). In *Arabidopsis*, the presence of CHG methylation and H3K9me2 over promoter regions is associated with transcriptional silencing [42]–[43]. Although the average profile shows very low levels of these marks near the TSS there are some genes that exhibit enrichment for CHG methylation and/or H3K9me2 in the 100 bp tile that overlaps the TSS. There are 459 maize FGS genes that have high H3K9me2 (>1 standard deviation above genome-wide average) and 546 FGS genes that had high CHG methylation (>88.5%) in the 100 bp tile that overlaps the TSS (Table S1). Although there is a relatively small overlap in these two sets of genes (Figure 5B) there was evidence that both marks tended to be enriched at most of these genes (Figure 5C). In many cases only one mark met the stringent criteria for discovery but the other mark was also elevated (Figure 5A). In contrast to genes containing transposons, the relative expression of genes with high CHG or H3K9me2 appeared to be strongly depressed relative to all genes (Figure 5A). The expression patterns for these genes were investigated in the developmental atlas representing 60 different tissues or organs of B73 [44]. Many of these genes (45% of high CHG TSSs and 53% of high H3K9me2 TSSs) were not detected in any of the 60 tissues with RNA-seq data. Only 33 of the high CHG TSS genes and 22 of the high H3K9me2 TSS genes exhibit expression levels over 10 FPKM (Fragments Per Kilobase per Million). A small subset of these genes exhibit tissue-specific expression (Figure S6, S7). The genes with elevated CHG or H3K9me2 at the TSS often did not have homology to genes in other grass species (Table S1). Many of these sequences may represent mis-annotated sequences that are not functional genes.

## Discussion

Although the genome-wide patterns of DNA methylation and H3K9me2 in maize and *Arabidopsis* are generally similar there are several interesting differences. One of the most notable differences in the pattern of DNA methylation is observed in regions surrounding maize and *Arabidopsis* genes. While *Arabidopsis* genes are generally flanked by regions with low levels of DNA methylation and H3K9me2 maize genes are flanked by regions with elevated levels for these marks. This is likely due to the interspersed organization of genes and transposons within the maize genome [12], [14]. The majority of transposons in the *Arabidopsis* genome are found within percentromeric regions of knob-like heterochromatin structures. In maize, transposons are found throughout the chromosome, interspersed with genes.

The presence of CHG methylation and H3K9me2 within maize gene bodies is somewhat unexpected. One source of CHG methylation and H3K9me2 within maize gene bodies is the presence of transposons within introns. Although long introns containing heterochromatic sequence are common in animal genomes they are relatively rare in *Arabidopsis*. Only ∼130 *Arabidopsis* genes contain long introns with elevated levels of CHG methylation [41]. However, these are much more common within the rice genome [41]. Here we show that these long introns, decorated with CHG methylation and H3K9me2, are also present in thousands of maize genes. These introns generally contain transposon insertions. The transposons insertion located within introns contain levels of CG, CHG and H3K9me2 methylation similar to that observed for transposons located elsewhere in the genome and the levels of these modifications are not influenced by the expression level of the gene itself. The genes containing these insertions show a full range of expression levels similar to that observed for other maize genes suggesting that the presence of a region containing CHG and H3K9me2 does not pose substantial barrier to transcriptional elongation. There is evidence that allelic variation for the insertion of a heavily methylated retrotransposon does not result in differences in transcript abundance at the *Zmet2* locus [45]. In *Arabidopsis*, the *IBM2/ASI1* gene is required for the ability to properly transcribe through introns containing CHG methylation [20], [41]. Orthologs of this gene exist in maize and likely are required for active transcription through introns containing CHG and H3K9me2 methylation.

Maize has lower average levels of CHH methylation than *Arabidopsis*. The CHH methylation that is observed in *Arabidopsis* is found at many different transposons. In *Arabidopsis*, CHH methylation can be due to RdDM-targeting of the DRM enzymes [1] or by the CMT2 gene which seems to be targeted to regions containing H3K9me2 [10] and requires DDM1 [5]. While maize does contain DRM genes [46] and chromomethylases [47], there is no evidence for orthologs of CMT2 in maize [5]. The lack of a CMT2 in maize could explain the lack of elevated CHH methylation levels within retrotransposons that are heavily silenced by H3K9me2. The analysis of H3K9me2 and CHH levels in maize TE families reveals that only one of these two marks is usually enriched in each family (Figure 2D–E). Since a large portion of the maize genome is derived from retrotransposons and these sequences tend to have very low levels of CHH methylation in maize the genome-wide levels of CHH methylation are quite low in maize. The observation that only certain TIR families contain high levels of CHH methylation in maize is intriguing. These TIR families (DTM and DTC) are enriched for being located near genes (average distance to nearest gene is under 3 kb) compared to the other TIR families (average distance to nearest gen is over 6 kb) which may indicate a preference for insertion in euchromatin. This may allow these elements to by targeted by the RdDM pathway while the other TIR families with insertions in non-genic regions would not be accessible for this pathway and would be silenced by H3K9me2.

*Arabidopsis* provides an excellent model system for studying the mechanisms that control the distribution of chromatin modifications in plant genomes. However, the relatively simple genome organization in *Arabidopsis* is not common in many plant species. Most plant species, including many crops, contain genomes with more complex organizations and the analysis of the epigenome in these plant species is likely to reveal important differences in the distribution of chromatin modifications.

## Supporting Information

Figure S1 Distribution of DNA methylation and H3K9me2 levels. (A) The accession numbers for each of the datasets used in this study is listed. The proportion of 100 bp tiles with varying levels of CG (B), CHG (C) or CHH (D) is shown for all regions, genic regions and TE regions. (E) The distribution of read counts (per 100 bp tile) is shown for all tiles, genic tiles and TE tiles.
(TIF)

Figure S2 Relative distance line plots across sub-families of maize transposable elements. *Zea mays* transposable elements were split by their different sub-families and aligned at their 3′ and 5′ ends. Distance across the transposable elements was normalized to a scale of 1 to 1000. Average percent methylation and H3K9me2 reads at each distance are displayed.
(TIF)

Figure S3 DNA methylation profiles of different types of maize genes. (A) The relative levels of DNA methylation in each context or H3K9me2 ChIP-seq read counts are plotted for all maize genes (black), the filtered gene set (FGS-red) and the working gene set (WGS-green). (B) Maize genes were split by their classification as either syntenic or inserted [35] and aligned at their 3′ and 5′ ends. The average DNA methylation within either exons or introns is shown. (C) The genes were classified as either sub-genome1 or sub-genome 2 [38], aligned at their 3′ and 5′ ends and methylation levels in each context are plotted.
(TIF)

Figure S4 Additional examples of transposable elements located within genes. Genic transposable elements as viewed in Integrated Genomics Viewer (IGV) [48]. H3K9me2 reads are displayed in red; transposable elements in pink; CG, CHG, and CHH methylation are represented as percent methylation across 100 bp tiles.
(TIF)

Figure S5 Absolute distance line plots of DNA methylation and H3K9me2 over genic space in maize and *Arabidopsis*. Maize and *Arabidopsis* genes were aligned at the 5′ and 3′ ends and CG (A), CHG (B) and CHH (C) DNA methylation levels or H3K9me2 read counts (D) are plotted. The vertical dashed lines represent the 5′ and 3′ ends. The regions within genes are classified as introns (red), exons (black) or introns with TEs masked (blue). (E) The proportion of TEs (>1,000 bp) located within maize genes that are annotated as TIR, LINE or LTR elements is shown compared to the proportion of all TEs in the maize genome in each of these three classes.
(TIF)

Figure S6 Clustering of expression levels for genes with high CHG methylation in promoter regions. Many of these genes show very low levels of expression. There are ∼80 of these genes with low levels of expression (1-5FPKM) in a large number of tissues. There are only 4 genes with high expression levels (at least 100FPKM). Two of these genes show anther specific expression and the other two exhibit expression in specific leaf tissues.
(TIF)

Figure S7 Clustering of expression levels for genes with high H3K9me2 in promoter regions. The majority of these genes show very low levels of expression. There are 40 of these genes with low levels of expression (1-5FPKM) in a large number of tissues. There are about 10 genes that show high levels of expression (>100FPKM) in at least one tissue. Four of these genes show anther specific expression and four show endosperm specific expression while the last two have leaf-specific expression.
(TIF)

Table S1 Genes with High H3K9me2 and/or high CHG methylation over transcription start site.
(XLSX)
