# Supplementary figures and images for: Genomic Distribution of H3K9me2 and DNA Methylation in a Maize Genome
Source: PLoS One. 2014 Aug 14;9(8):e105267. doi: 10.1371/journal.pone.0105267 (PMC4133378; doi:10.1371/journal.pone.0105267)

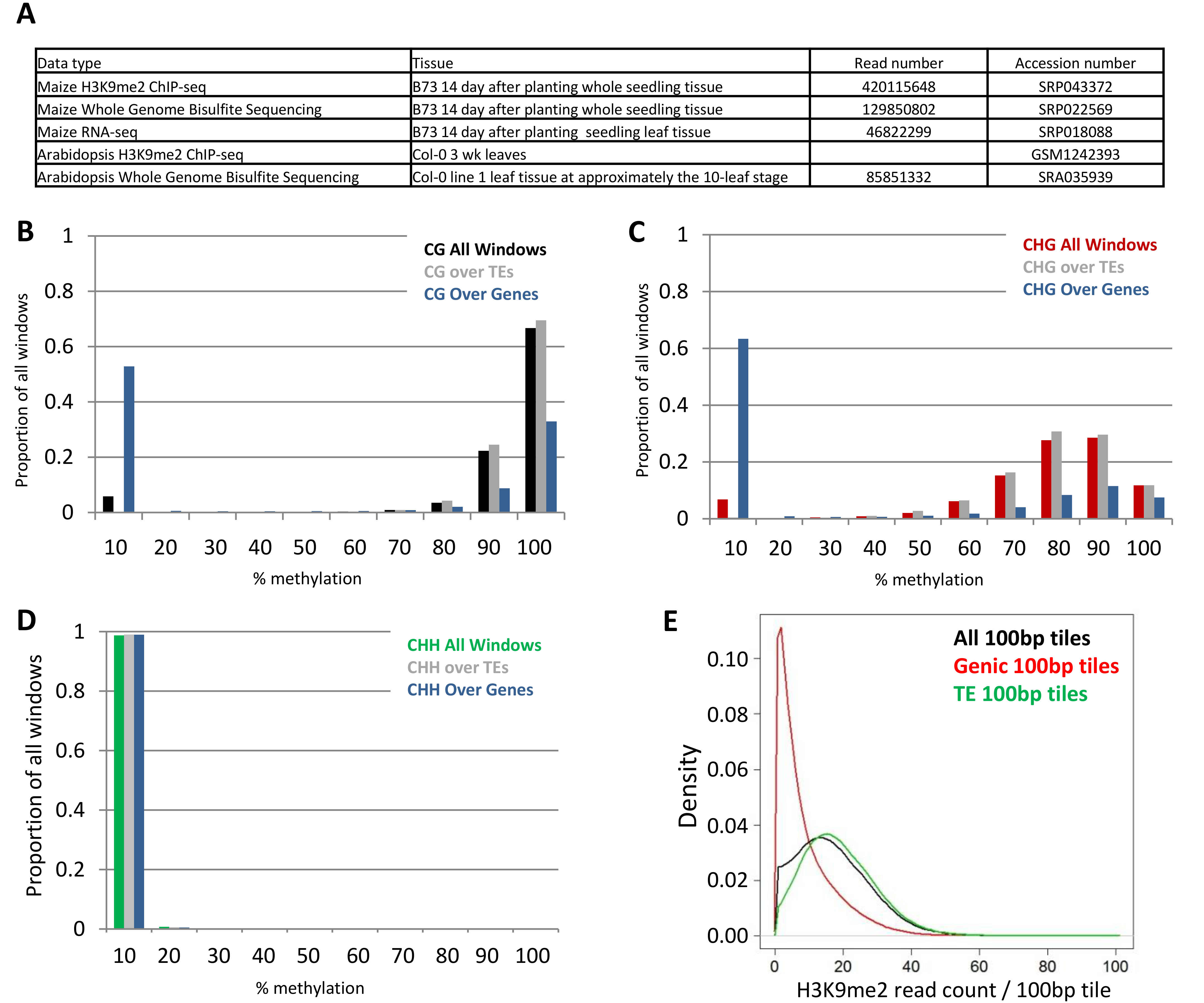

Supplement: Figure S1 — Distribution of DNA methylation and H3K9me2 levels. (A) The accession numbers for each of the datasets used in this study is listed. The proportion of 100 bp tiles with varying levels of CG (B), CHG (C) or CHH (D) is shown for all regions, genic regions and TE regions. (E) The distribution of read counts (per 100 bp tile) is shown for all tiles, genic tiles and TE tiles. (TIF) [file pone.0105267.s001.tif]

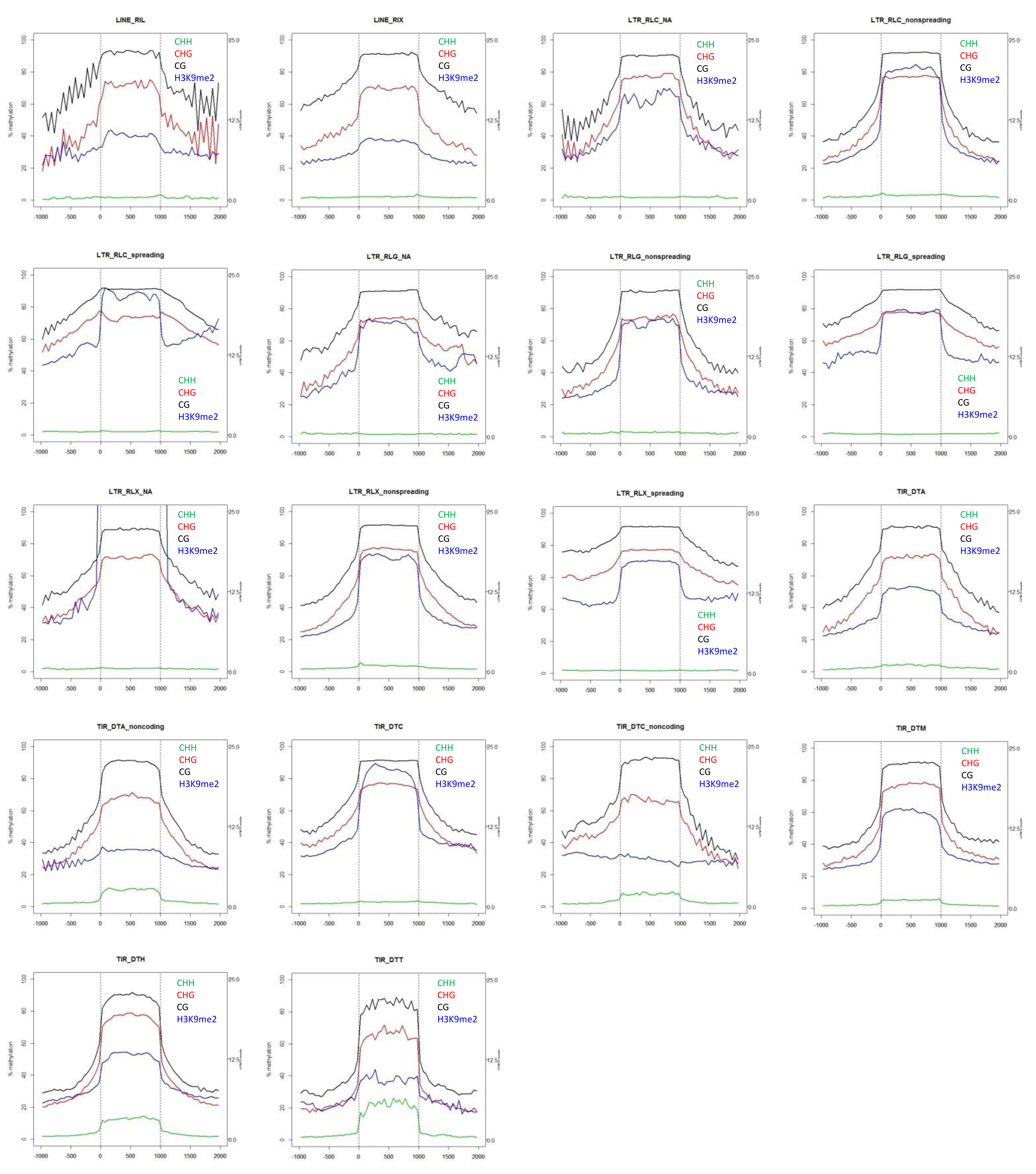

Supplement: Figure S2 — Relative distance line plots across sub-families of maize transposable elements. Zea mays transposable elements were split by their different sub-families and aligned at their 3′ and 5′ ends. Distance across the transposable elements was normalized to a scale of 1 to 1000. Average percent methylation and H3K9me2 reads at each distance are displayed. (TIF) [file pone.0105267.s002.tif]

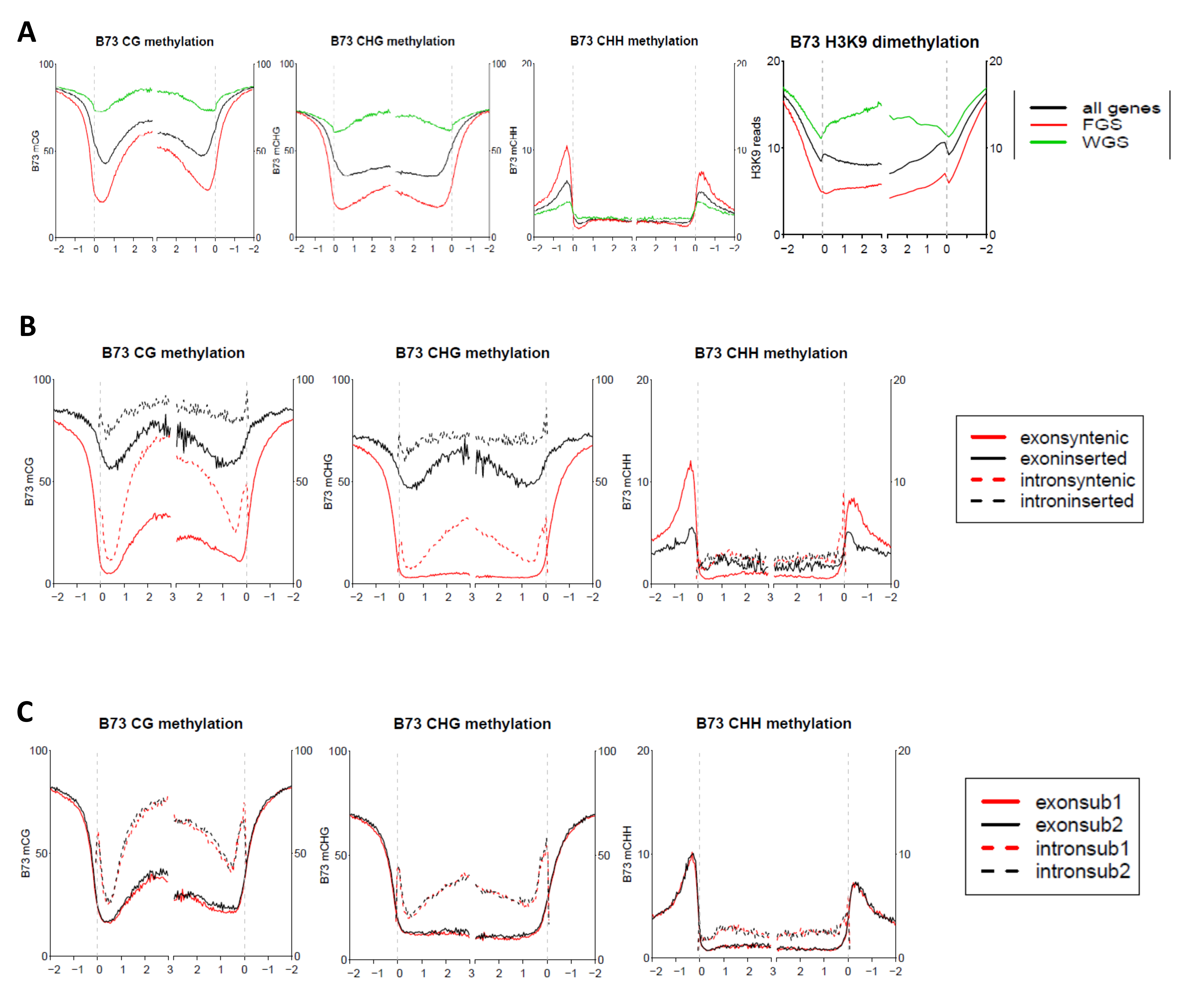

Supplement: Figure S3 — DNA methylation profiles of different types of maize genes. (A) The relative levels of DNA methylation in each context or H3K9me2 ChIP-seq read counts are plotted for all maize genes (black), the filtered gene set (FGS-red) and the working gene set (WGS-green). (B) Maize genes were split by their classification as either syntenic or inserted [35] and aligned at their 3′ and 5′ ends. The average DNA methylation within either exons or introns is shown. (C) The genes were classified as either sub-genome1 or sub-genome 2 [38], aligned at their 3′ and 5′ ends and methylation levels in each context are plotted. (TIF) [file pone.0105267.s003.tif]

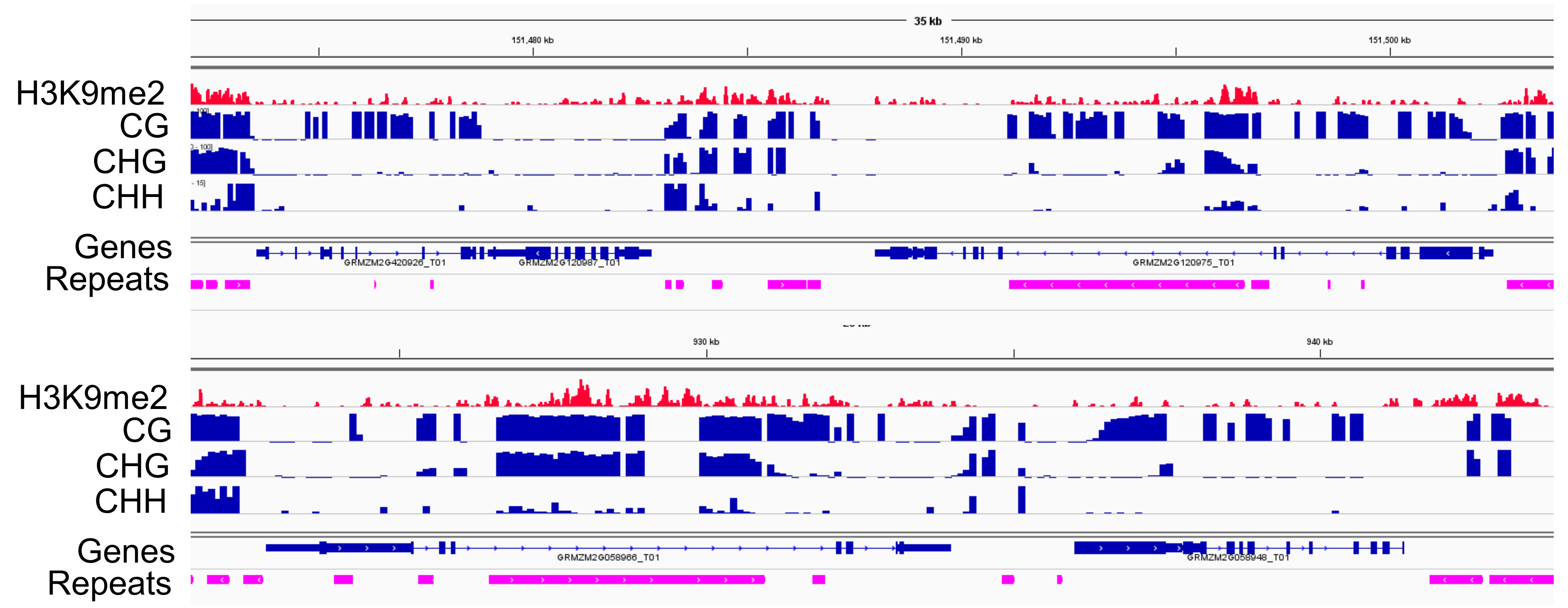

Supplement: Figure S4 — Additional examples of transposable elements located within genes. Genic transposable elements as viewed in Integrated Genomics Viewer (IGV) [48]. H3K9me2 reads are displayed in red; transposable elements in pink; CG, CHG, and CHH methylation are represented as percent methylation across 100 bp tiles. (TIF) [file pone.0105267.s004.tif]

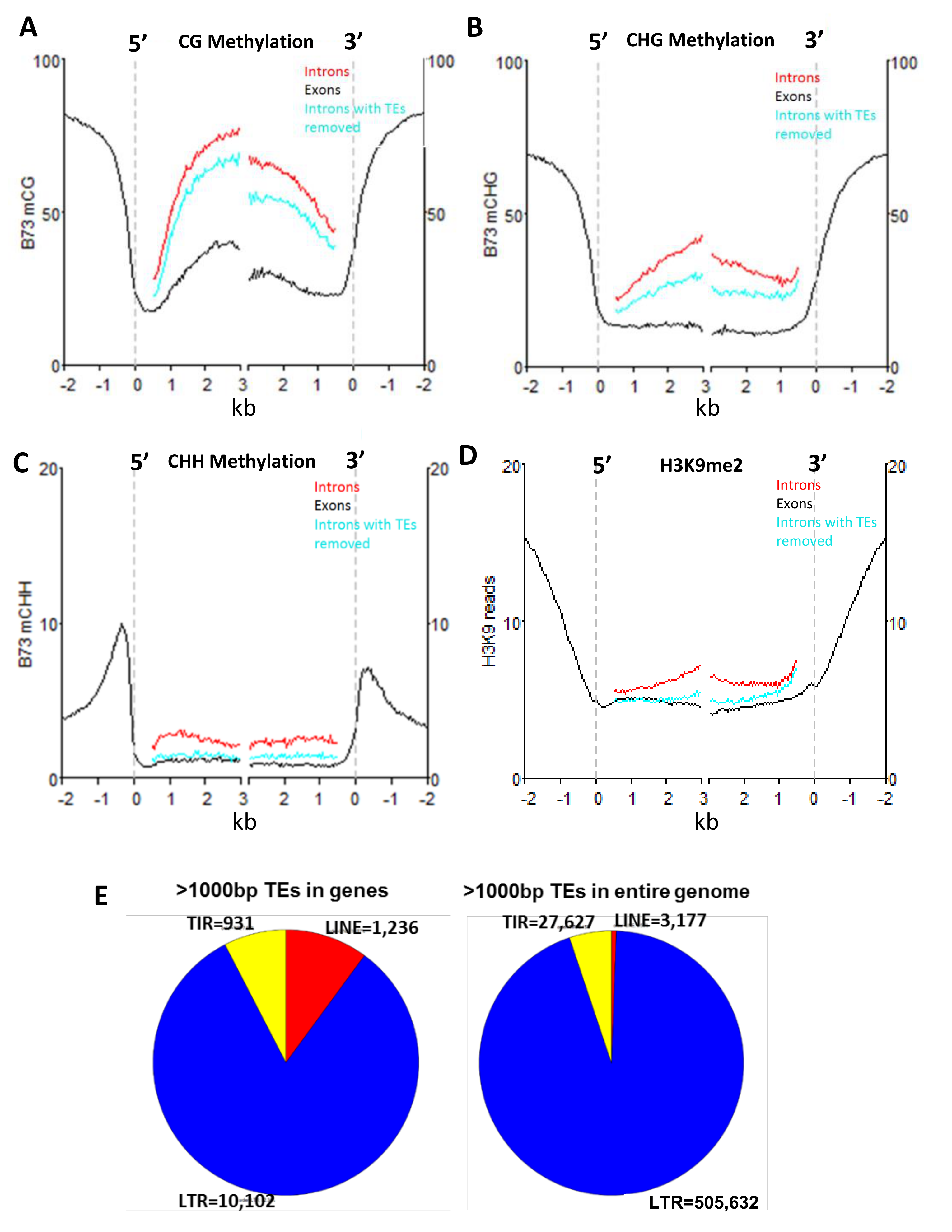

Supplement: Figure S5 — Absolute distance line plots of DNA methylation and H3K9me2 over genic space in maize and Arabidopsis. Maize and Arabidopsis genes were aligned at the 5′ and 3′ ends and CG (A), CHG (B) and CHH (C) DNA methylation levels or H3K9me2 read counts (D) are plotted. The vertical dashed lines represent the 5′ and 3′ ends. The regions within genes are classified as introns (red), exons (black) or introns with TEs masked (blue). (E) The proportion of TEs (>1,000 bp) located within maize genes that are annotated as TIR, LINE or LTR elements is shown compared to the proportion of all TEs in the maize genome in each of these three classes. (TIF) [file pone.0105267.s005.tif]

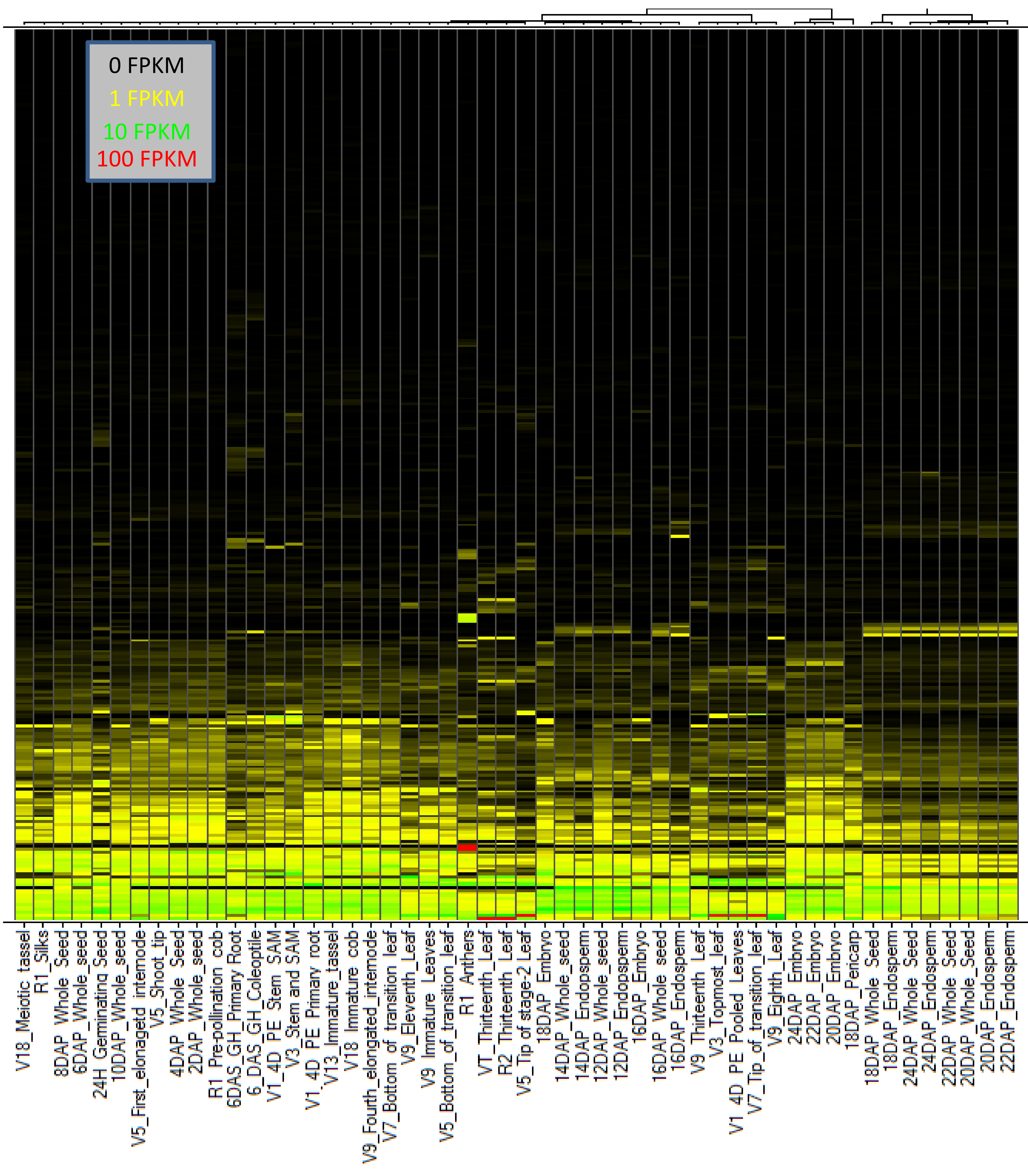

Supplement: Figure S6 — Clustering of expression levels for genes with high CHG methylation in promoter regions. Many of these genes show very low levels of expression. There are ∼80 of these genes with low levels of expression (1-5FPKM) in a large number of tissues. There are only 4 genes with high expression levels (at least 100FPKM). Two of these genes show anther specific expression and the other two exhibit expression in specific leaf tissues. (TIF) [file pone.0105267.s006.tif]

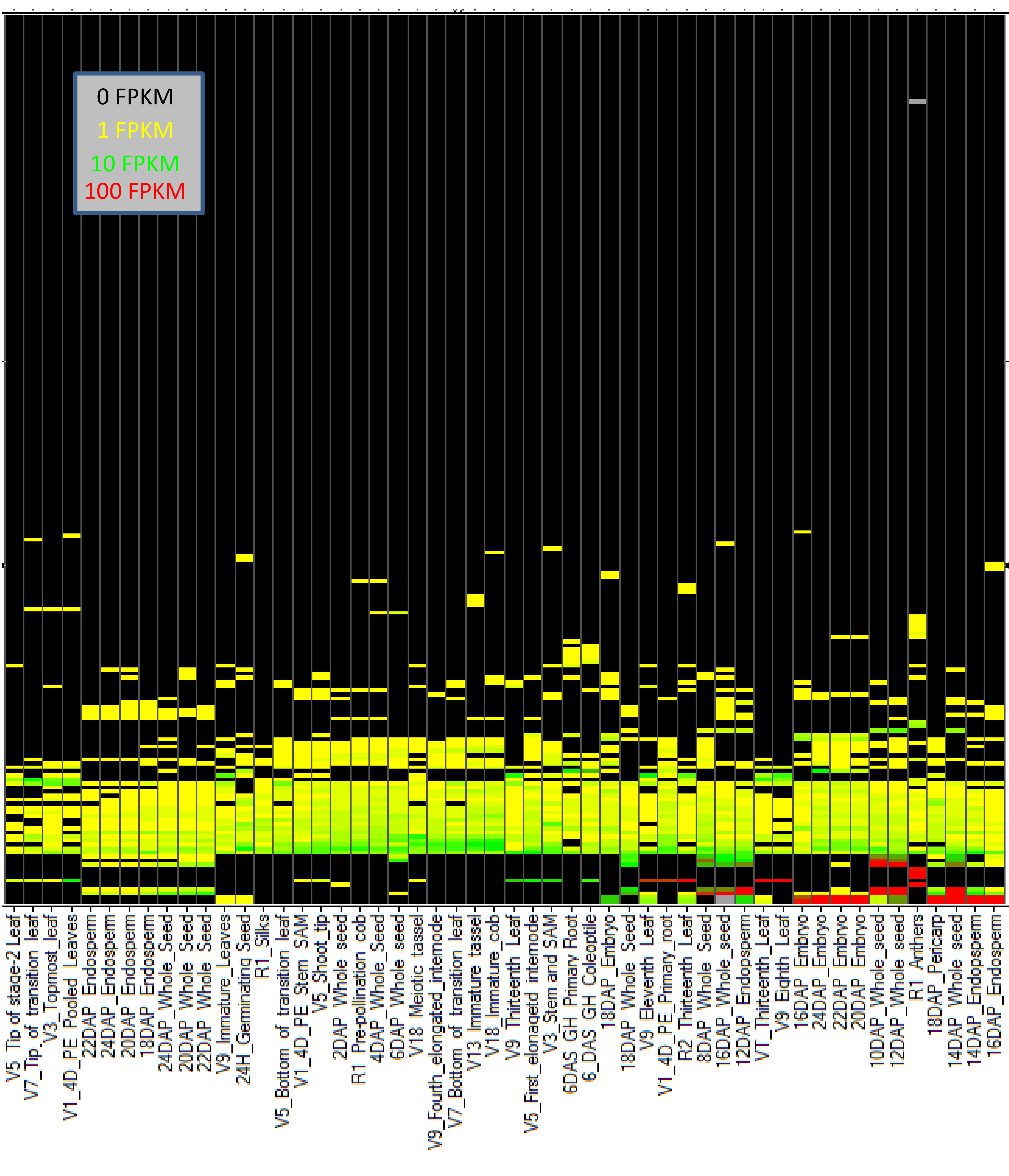

Supplement: Figure S7 — Clustering of expression levels for genes with high H3K9me2 in promoter regions. The majority of these genes show very low levels of expression. There are 40 of these genes with low levels of expression (1-5FPKM) in a large number of tissues. There are about 10 genes that show high levels of expression (>100FPKM) in at least one tissue. Four of these genes show anther specific expression and four show endosperm specific expression while the last two have leaf-specific expression. (TIF) [file pone.0105267.s007.tif]
